# Everolimus-Induced Immune Effects after Heart Transplantation: A Possible Tool for Clinicians to Monitor Patients at Risk for Transplant Rejection

**DOI:** 10.3390/life11121373

**Published:** 2021-12-10

**Authors:** Kristin Klaeske, Sven Lehmann, Robert Palitzsch, Petra Büttner, Markus J. Barten, Khalil Jawad, Sandra Eifert, Diyar Saeed, Michael A. Borger, Maja-Theresa Dieterlen

**Affiliations:** 1 Heart Center, Department of Cardiac Surgery, HELIOS Clinic, University Hospital Leipzig, Strümpellstraße 39, 04289 Leipzig, Germany; kristin.klaeske@helios-gesundheit.de (K.K.); Sven.lehmann2@gesundheitnord.de (S.L.); robert.palitzsch@krankenhaus-muldental.de (R.P.); Khalil.jawad@medizin.uni-leipzig.de (K.J.); Sandra.Eifert@helios-gesundheit.de (S.E.); diyar.saeed@helios-gesundheit.de (D.S.); Michael.Borger@helios-gesundheit.de (M.A.B.); 2 Heart Center Leipzig, Department of Internal Medicine and Cardiology, University of Leipzig, Strümpellstraße 39, 04289 Leipzig, Germany; Petra.Buettner@medizin.uni-leipzig.de; 3 Department of Cardiovascular Surgery, University Heart and Vascular Center Hamburg, Martinistraße 52, 20246 Hamburg, Germany; m.barten@uke.de

**Keywords:** everolimus, heart transplantation, tolerance induction, rejection, regulatory T cells, dendritic cells, flow cytometry

## Abstract

Background: Patients treated with an inhibitor of the mechanistic target of rapamycin (mTORI) in a calcineurin inhibitor (CNI)-free immunosuppressive regimen after heart transplantation (HTx) show a higher risk for transplant rejection. We developed an immunological monitoring tool that may improve the identification of mTORI-treated patients at risk for rejection. Methods: Circulating dendritic cells (DCs) and regulatory T cells (T_regs_) were analysed in 19 mTORI- and 20 CNI-treated HTx patients by flow cytometry. Principal component and cluster analysis were used to identify patients at risk for transplant rejection. Results: The percentages of total T_regs_ (*p* = 0.02) and CD39^+^ T_regs_ (*p* = 0.05) were higher in mTORI-treated patients than in CNI-treated patients. The principal component analysis revealed that BDCA1^+^, BDCA2^+^ and BDCA4^+^ DCs as well as total T_regs_ could distinguish between non-rejecting and rejecting mTORI-treated patients. Most mTORI-treated rejectors showed higher levels of BDCA2^+^ and BDCA4^+^ plasmacytoid DCs and lower levels of BDCA1^+^ myeloid DCs and T_regs_ than mTORI non-rejectors. Conclusion: An mTORI-based immunosuppressive regimen induced a sufficient, tolerance-promoting reaction in T_regs_, but an insufficient, adverse effect in DCs. On the basis of patient-specific immunological profiles, we established a flow cytometry-based monitoring tool that may be helpful in identifying patients at risk for rejection.

## 1. Introduction

In patients undergoing heart transplantation (HTx), efficient immunosuppression is still a major goal in the post-HTx period. The clinical introduction of inhibitors of the mechanistic target of rapamycin (mTORIs) in a calcineurin inhibitor (CNI)-free triple-drug immunosuppressive regimen for HTx patients is accompanied by adverse effects. Clinical multi-centre trials have demonstrated an increased risk for acute cellular rejection (ACR), mortality, and severe infections during CNI-free mTORI-based triple-drug immunosuppression after HTx [1,2]. The SCHEDULE trial showed that CNI withdrawal after a quadruple immunosuppressive regimen consisting of CNI, the mTORI everolimus (ERL), mycophenolate mofetil (MMF), and steroids could improve renal function and significantly reduce the progression of cardiac allograft vasculopathy (CAV) [3]. The MANDELA trial demonstrated beneficial effects on renal function and a higher rejection rate in ERL-treated, CNI-free patients in comparison to patients with a CNI-reduced treatment [4]. Additionally, CNI reduction in ERL- and steroid-treated HTx patients was reported to reduce the rate of cytomegalovirus (CMV) infection [5]. There is consensus that some patient populations benefit from mTORI-based immunosuppressive regimens, including (i) de novo HTx patients with a high risk for CMV infection or early CAV, (ii) de novo HTx patients with renal insufficiency, (iii) patients who require an immunosuppressive conversion due to renal insufficiency, and (iv) patients who develop skin cancer [6].

Because CNI-free mTORI-treated patients have a higher risk for transplant rejection [3,7], this cohort could benefit from an immunological monitoring tool that identifies patients with an immunological profile promoting transplant rejection. To consider the immunological state in addition to mTORI blood levels could improve the safety of the immunosuppression in these patients. Regulatory T cells (T_regs_) and distinct subsets of myeloid (mDCs) and plasmacytoid dendritic cells (pDCs) are known to be involved in the prevention of allograft rejection [8]. Therefore, the aim of the present study was to develop an immunological monitoring tool for an improved identification of mTORI-treated patients at risk for transplant rejection. The immunological monitoring tool included the evaluation of cell subsets that are known to be involved in the prevention of allograft rejection following HTx.

## 2. Material and Methods

### 2.1. Study Groups and Patient Characteristics

This study was approved by the Institutional Review Board of the Medical Faculty from the University of Leipzig, Germany (vote number: 405/14ek). The patients were informed about the aims of the study prior to study initiation. All patients gave written informed consent.

The study included 19 HTx patients with mTORI-based immunosuppression and 20 HTx patients with CNI-based immunosuppression who received HTx between February 2007 and January 2015. The HTx had been conducted at least 12 months before study initiation. In cases involving immunosuppressive conversions, study entry was allowed six months after conversion to avoid wash-out effects. The clinical characteristics of patients, including their age at study initiation and at HTx, sex, body mass index (BMI), diagnosis leading to HTx listing, re-HTx status, CMV positivity, ventricular assist device implantation before HTx, the initial immunosuppression, the immunosuppression at study initiation and comorbidities, were documented.

### 2.2. Blood Sampling

Peripheral heparinized whole-blood samples were collected during the outpatient visits in the clinic and immediately analysed by flow cytometry.

### 2.3. Flow Cytometric Analysis of DC and T_reg_ Subpopulations

The experimental procedures were performed as described previously [9]. T_regs_ were defined as CD3^+^CD4^+^CD25^high^CD127^low^ cells and peripheral blood DCs as HLA^+^ and lineage cocktail (lin)-1^−^ cells. Functionally characterized T_reg_ and DC subsets known to be involved in transplant tolerance induction were chosen for this study.

We analysed the T_reg_ subsets expressing CD39, CD62L, CD120b, and CD147 as well as the subsets of DCs expressing blood dendritic cell antigens (BDCAs) 1, 2, 3 or 4. For immunological staining of the DC and T_reg_ subpopulations, whole-blood samples were incubated with different fluorescence-labelled antibodies for 20 min at 21 °C. DCs and their subpopulations were stained using the antibodies lin-1-FITC, HLA-DR-PerCP, BDCA2-PE, BDCA4-APC, BDCA1-PE, and BDCA3-APC. CD3-PerCP/Cy5.5, CD4-APC/H7, CD25-PE/Cy7, CD127-APC, CD120b-PE, CD147-FITC, CD62L-PE, and CD39-FITC were used for staining T_regs_ and their subsets. All antibodies were purchased from BD Biosciences (Heidelberg, Germany) and from BioLegend (San Diego, CA, USA). After incubation with the antibodies, red blood cells were lysed with FACS lysing solution (BD Biosciences, Franklin Lakes, NJ, USA) for 10 min. Cells were centrifuged at 300× *g* for 5 min at 21 °C and washed with 4 mL of phosphate-buffered saline. After an additional centrifugation, cells were fixed with 500 µL of 1% formalin in phosphate-buffered saline before analysis. Flow cytometric analysis was performed using a BD Biosciences LSR II cytometer with FACS Diva 6.1.3 software (both from BD Biosciences, Franklin Lakes, NJ, USA). Standardization of the instrument was performed by measurements of *Cytometer Setup and Tracking Beads* (BD Biosciences) once a week. At least 10,000 total DCs and T_regs_ were measured per sample and panel, and the mean fluorescence intensities (MFIs) were documented.

### 2.4. Principle Component Analysis

We combined immune markers that are involved in the prevention of ACR and tolerance induction to describe the patient’s immune transplant tolerance phenotype. The immune phenotype was defined on the basis of the percentages of tolerance-inducing immune cells and was called immunological profile. Principal component analysis (PCA) was performed using R Studio Version 1.0.143 and the packages factoextra version 1.0.5 (written by Alboukadel Kassambara and Fabian Mundt; both RStudio, Boston, MA, USA) and ggplot2 version 2.2.1 (written by Hadley Wickham, Winston Chang, Lionel Henry, Thomas Lin Pedersen, Kohske Takahashi, Claus Wilke, and Kara Woo; RStudio). A biplot was created using the two dimensions contributing to the highest variance proportion. Five flow cytometric parameters of DCs (% total DCs/peripheral blood mononuclear cells (PBMCs), % BDCA1^+^ DCs/total DCs, % BDCA2^+^ DCs/total DCs, % BDCA3^+^ DCs/total DCs and % BDCA4^+^ DCs/total DCs) and six parameters of T_regs_ (% CD4^+^ T cells/total T cells, % T_regs_/CD4^+^ T cells, % CD39^+^ T_regs_/total T_regs_, % CD62L^+^ T_regs_/total T_regs_, % CD120b^+^ T_regs_/total T_regs_ and % CD147^+^ T_regs_/total T_regs_) have been included in the PCA.

Parameters showing the highest contribution for discriminating between the immunological profiles of mTORI- and CNI-treated HTx patients in PCA were visualized in a clustered heatmap. The ClustVis program (Bioinformatics, Algorithms & Data Mining Group, University of Tartu, Tartu, Estonia) was used to create heatmaps. The hierarchical cluster analysis allows pattern recognition of a tolerance-inducing phenotype and displays the distance connectivity of the immunological profile for every patient. This made it possible to monitor whether an HTx patient tends to have a tolerance-promoting immunological phenotype or if the immunological profile shifts to that of rejecting patients. The monitoring tool could be helpful for clinicians to decide if a patient has an increased risk for rejection according to his/her immunological profile. Patient-specific results can be obtained five hours following blood withdrawal.

### 2.5. Statistical Analyses

Statistical data analyses were performed using SPSS Statistics version 25 (IBM Corp., Armonk, NY, USA 1989, 2017). Metric data are presented as mean (± standard deviation) for continuous variables and by the value (percent) for categorical variables, unless stated otherwise. An unpaired t-test was used for residuals showing a normal distribution and for two-group comparisons of metric variables. For all analyses, two-sided tests were performed at a *p* ≤ 0.05 significance level. 

## 3. Results

We compared two patient groups with different immunosuppressive regimens: HTx patients undergoing CNI-based immunosuppression (*n* = 20) and HTx patients treated with an mTORI-based immunosuppression (*n* = 19). Because CNI- and mTORI-based immunosuppression induce different immunological effects that are associated with the risk of rejection, we compared both groups in relation to their cell subsets known to be relevant in transplant immunology. The aim of this comparison was the identification of the mTORI-specific changes of the immune system.

### 3.1. Patient Characteristics

Both groups were comparable with respect to the age at HTx, age at study initiation, sex, BMI, CMV positivity, and diagnoses leading to HTx, which was predominantly dilative cardiomyopathy (Table 1). Patients of both groups showed a similar rejection rate (*p* = 0.14), which was defined as biopsy-proven ACR of grade ≥1 B according to the International Society of Heart and Lung Transplantation grading system.

The initial immunosuppression and immunosuppressive therapy at study initiation are shown in Table 1. Furthermore, the comorbidities in mTORI- and CNI-treated patients at study initiation were comparable (Table 2).

### 3.2. Immunological Group Comparison

Intergroup comparisons of T_reg_ and DC subsets such as mDCs and pDCs, which play major roles in transplant tolerance and are therefore potential markers for patients at a higher risk of ACR after HTx, were performed. Our data revealed that the percentage of total DCs among PBMCs, the percentages of BDCA1^+^ mDCs, BDCA3^+^ mDCs, BDCA2^+^ pDCs, and BDCA4^+^ pDCs as well as the MFIs of BDCA1-4 were comparable between the CNI- and the mTORI groups (Table 3).

The percentages of CD4^+^ T helper-cells, CD120b^+^, CD147^+^, and CD62L^+^ T_regs_ as well as the MFIs of CD147^+^, CD120b^+^, CD39^+^ and CD62L^+^ T_regs_ were comparable in both groups, whereas the percentages of total T_regs_ and CD39^+^ T_regs_ were significantly higher in mTORI-treated patients (Table 3).

### 3.3. Creating a Monitoring Tool

Because previous reports showed that CNI- and mTORI-treated patients differ in relation to their rejection rates, we hypothesized differences in their immunological status. Therefore, a PCA was performed using a dataset consisting of 11 flow cytometric parameters.

**Figure 1:**
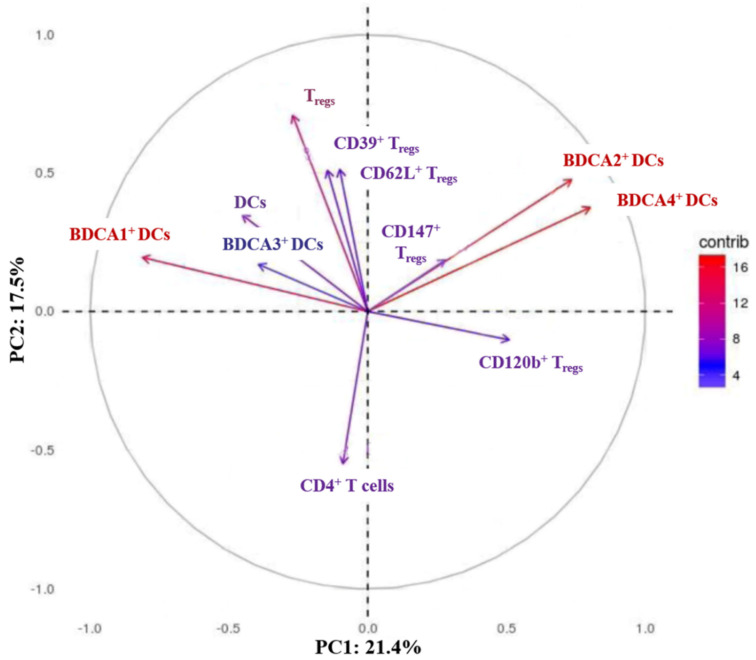
Biplot of the two principal components contributing to the highest variability in a dataset of 11 cellular markers identified using a principal component analysis. This plot illustrates the influence of total DCs, DC subsets, CD4^+^ T cells, T_regs_, and their subpopulations on the discrimination between CNI- and mTORI-treated patients after HTx. Score plots of PC1 and PC2 were visualized for all measured cellular parameters. The contribution to variability range was indicated by different colours (blue = low contribution, red = high contribution). Note: BDCA1/2/3/4, blood dendritic cell antigen 1/2/3/4; CD, cluster of differentiation; CNI, calcineurin inhibitor; DCs, dendritic cells; mTORI, inhibitor of the mechanistic target of rapamycin; PC, principle component; T_regs_, regulatory T cells.

The first and second principal components visualized in the biplot in Figure 1 explained 24.1% and 17.5%, respectively, of the total variation in the flow cytometric data between CNI- and mTORI-treated patients. The analysis identified a strong influence of total T_regs_ and BDCA1-, BDCA2-, and BDCA4-expressing DCs in discriminating between CNI and mTORI patients. Total DCs and CD4^+^ T helper cells showed a moderate ability to discriminate between the two groups. BDCA3^+^ DCs and CD62L^+^, CD39^+^, CD147^+^, and CD120b^+^ T_regs_ were less capable of discriminating between the two groups.

**Figure 2:**
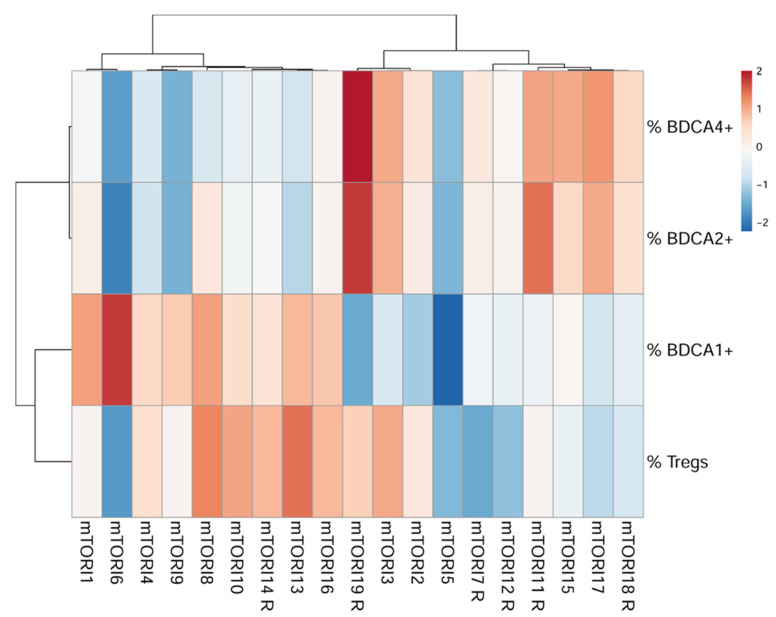
Clustered heat map based on differentially abundant immunological cell subsets of BDCA1^+^, BDCA2^+^, BDCA4^+^ DCs and total T_regs_ from a dataset of 19 HTx patients receiving mTORI-based immunosuppression. The colour scale indicates the expression in comparison with the mean of all patients (blue: lower expression compared to the mean, red: higher expression compared to the mean). Note: BDCA1/2/3/4, blood dendritic cell antigen 1/2/3/4; mTORI, inhibitor of the mechanistic target of rapamycin; mTORIx R, rejecting patient with an immunosuppressive regimen containing an inhibitor of the mechanistic target of rapamycin; T_regs_, regulatory T cells.

After PCA, clustered heatmaps including the parameters with the strongest influence (BDCA1^+^ mDCs, BDCA2^+^ pDCs, BDCA4^+^ pDCs, and total T_regs_) were separately generated for mTORI-treated patients. In the mTORI group, one cluster included 89% (*n* = 8) of the mTORI-treated patients without rejection and 11% (*n* = 1) of the mTORI-treated patients with rejection episodes (Figure 2). These patients were characterized by lower percentages of BDCA2^+^ and BDCA4^+^ pDCs and higher percentages of BDCA1^+^ mDCs and total T_regs_. The second cluster included 50% (*n* = 5) mTORI patients without rejection and 50% (*n* = 5) mTORI patients with rejection. The patients of the second cluster had a higher percentage of BDCA2^+^ DCs and BDCA4^+^ pDCs as well as a lower percentage of BDCA1^+^ mDCs and total T_regs_ than the patients of the first cluster.

## 4. Discussion

In this study, we compared the effect of CNI- and mTORI-based immunosuppression on immune cells to identify the immunological differences that result from mTORI-treatment. We found that mTORI-based immunosuppression is associated with higher percentages of total T_regs_ and highly suppressive CD39^+^ T_reg_ subpopulations. In a second step, we established a monitoring tool based on the measurement of four immune cell subsets that may be helpful for identifying mTORI-patients with an increased allograft rejection risk.

T_regs_ are a subset of CD4^+^ T cells and participate in tolerance induction by suppressing immunological cell subsets such as T- and B-lymphocytes, DCs, monocytes and granulocytes [10,11]. The potential of T_regs_ to suppress the immune response by direct and indirect mechanisms may reinforce the finding that T_regs_ can prevent transplant rejection [12].

Although previous studies reported a negative influence of CNI-based immunosuppression on T_reg_ function and survival, studies investigating the effects of the mTORI rapamycin showed that the activity and homeostasis of T_regs_ was not affected to the same extent as those of conventional T cells [13]. In vivo studies after transplantation reported a pronounced increase in T_regs_ 3–6 months after conversion to an mTORI treatment [14,15]. In a non-human primate model, the repeated infusion of T_regs_ combined with mTORI treatment resulted in longer survival after allogeneic renal transplants [16]. In our study, mTORI treatment induced an increase in T_regs_ and CD39^+^ T_regs_ in comparison with CNI treatment. Similar findings highlighting an increase in T_regs_ have been reported in mTORI-treated renal transplants [15,17]. CD39^+^ T_regs_ have a highly suppressive capacity and belong to a special subset of memory T_regs_ [18,19]. Therefore, our data suggest that mTORI treatment leads to a higher immunosuppressive state of T_regs_ than CNI treatment, which should promote transplant tolerance. The findings of studies including renal transplant patients support our hypothesis and show that patients with renal allograft rejection had a reduced percentage of CD39^+^ T_regs_ [19,20].

The increased percentage of T_regs_ in mTORI-treated patients could explain the decreased rate of CAV after mTORI treatment [21,22]. Pilat and colleagues linked the immunosuppressive potency of T_reg_ cell therapy and CAV in a mouse model and showed that T_reg_ treatment potently avoids CAV [23]. However, an increase of T_regs_ alone seems to be insufficient for avoidance of ACR after transplantation, because CNI-free mTORI-based immunosuppression is associated with an increased risk for ACR [24].

T_regs_ are induced by DCs, which are antigen-presenting cells with critical roles in the induction and regulation of immunity [25]. In particular, BDCA2^+^ and BDCA4^+^ pDCs exert tolerogenic functions when mediating T_reg_ development and increasing T_reg_ frequencies [26,27]. Our data showed that the percentages of mDC and pDC subsets and MFI analysis of BDCA1-4 did not differ between mTORI- and CNI-treated patients. Previous studies reported that mTORIs are able to modulate DCs, while the stage of DC differentiation, the activation state as well as the duration and timing of DC exposure to mTORIs is crucial [8,25,28]. Furthermore, mTORIs are known to suppress maturation or induce apoptosis in conventional DCs and inhibit the activation of pDCs and cytokine production [29,30,31]. Therefore, we hypothesized that mTORI treatment may influence the distribution of BDCA1-4^+^ DC subsets.

An immune monitoring tool for clinical application in mTORI-treated patients is currently unavailable but desirable to identify patients at risk for transplant rejection. Our analysis revealed that evaluation of total T_regs_ and BDCA1^+^, BDCA2^+^, and BDCA4^+^ DCs could help to distinguish between rejecting and non-rejecting mTORI-treated patients. The identified cell subsets are known to be key players in induction processes and the maintenance of transplant tolerance. The outstanding role of T_regs_ in avoiding transplant rejection and promoting tolerance after transplantation has been proven. Additionally, the role of pDCs in mediating tolerance after transplantation has been reported [32,33,34]. In contrast, circulating BDCA1^+^ and BDCA3^+^ mDCs are more immunogenic than pDCs and show an increased capacity to process and present antigens via the major histocompatibility complex:peptide complex to CD4^+^ T cells, which could lead to the induction of an immune response when foreign antigens are presented [35].

In our cluster analyses, the cluster with a higher percentage of rejecting mTORI-treated patients showed higher levels of BDCA2^+^ and BDCA4^+^ pDCs and lower levels of BDCA1^+^ mDCs and T_regs_. We suggest that the mTORI treatment induced a sufficient, tolerance-promoting reaction in T_regs_, but an insufficient, adverse effect in DCs, which could explain the higher rejection rate of mTORI-treated patients in clinical studies. Monitoring results and their analysis could be available within five hours following blood withdrawal. Further, a sample storage up to 24 h at 21 °C is possible, which allows the transportation of blood samples from different hospitals to one analysis center.

This study was limited by its monocentric design and the small number of patients in each group. An increase in the patient number for heatmap analysis will strengthen the reliability of this analysis. Further, this study did not include patients with an immunosuppressive regimen combining mTORI and low-dose CNI. This group could be added in future studies to prove if the monitoring tool can detect patients at risk in an mTORI/low-dose CNI-treated cohort. Additionally, the study did not investigate differences in the functionality of T_regs_ and DCs among mTORI- and CNI-treated HTx patients.

## 5. Conclusions

In comparison with CNI-based immunosuppression, mTORI-based immunosuppression results in an immunological profile with significantly higher percentages of total T_regs_ and highly suppressive CD39^+^ T_reg_ subpopulations. On the basis of the patient-specific immunological profiles, we established a flow cytometry-based, four-parameter monitoring tool that may be helpful to identify patients at risk for allograft rejection receiving mTORI-based immunosuppression. This tool should be validated in a larger patient cohort to prove its enhancement of the safety of mTORI-treated HTx patients.

## Figures and Tables

**Table 1:** Patient characteristics and HTx-related clinical parameters in patients treated with mechanistic target of rapamycin-inhibitors (mTORI) or calcineurin-inhibitors (CNI).

	mTORI-Group *n* = 19	CNI-Group *n* = 20	*p* Value
age at HTx (years)	53.8 ± 9.5	52.9 ± 10.5	0.78
age at study begin (years)	59.7 ± 8.7	56.7 ± 10.9	0.96
male gender	14 (74%)	14 (70%)	0.80
BMI (kg/m^2^)	26.9 ± 4.8	28.0 ± 4.6	0.47
diagnosis leading to HTx-listing			0.62
ICM	6 (32%)	9 (45%)	
DCM	11 (58%)	10 (50%)	
congenital heart disease	2 (11%)	1 (5%)	
assist device prior HTx	6 (32%)	6 (30%)	0.92
rejection	6 (32%)	11 (55%)	0.14
CMV-positivity	0 (0%)	1 (5%)	0.32
initial IS therapy			0.14
TAC + MMF + GC	18 (95%)	14 (70%)	
CsA + MMF + GC	0 (0%)	3 (15%)	
CsA + ERL + GC	0 (0%)	2 (10%)	
ERL + MMF + GC	1 (5%)	1 (5%)	
IS therapy at study begin			
TAC + MMF + GC	-	15 (75%)	
CsA + MMF + GC	-	5 (25%)	
ERL + MMF + GC	15 (79%)	-	
ERL + MMF	4 (21%)	-	
trough level of IS at study begin ^#^	18 (100%) *	12 (60%)	<0.01

BMI, body mass index; CMV, cytomegalovirus; CsA, cyclosporine A; DCM, dilatative cardiomyopathy; ERL, everolimus; GC, glucocorticoid, HTx, heart transplantation; ICM, ischemic cardiomyopathy; IS, immunosuppression; MMF, mycophenolate-mofetil; TAC, tacrolismus; ^#^ target concentration: TAC 5–8 ng/mL, ERL 3–8 ng/mL, CsA 100–150 ng/mL; * serum level of one patient was missing.

**Table 2:** Comorbidity incidences in patients treated with mechanistic target of rapamycin-Inhibitors (mTORI) or calcineurin-inhibitors (CNI).

	mTORI-Group *n* = 19	CNI-Group *n* = 20	*p* Value
Hypertension	8 (42%)	14 (70%)	0.11
Cardiac arrhythmia	0 (0%)	2 (10%)	0.48
Pacemaker/ICD	4 (21%)	4 (20%)	1
COPD/Asthma bronchiale	1 (5%)	1 (5%)	1
Diabetes mellitus type 2	5 (26%)	6 (30%)	1
Hyperlipidemia	7 (37%)	10 (50%)	0.52
Hyperuricemia	4 (21%)	6 (30%)	0.72
Renal insufficiency *	11 (58%)	12 (60%)	1
grade 1	2 (11%)	0 (0%)	0.23
grade 2	3 (16%)	1 (5%)	0.34
grade 3	6 (32%)	10 (50%)	0.33
grade 4	0 (0%)	1 (5%)	1
Neurological diseases ^1^	3 (16%)	6 (30%)	0.45
Hematological diseases ^2^	0 (0%)	1 (5%)	1
Neoplasia ^3^	1 (5%)	4 (20%)	0.34
Chronic inflammation ^4^	2 (10%)	2 (10%)	1
Allergies	1 (5%)	3 (15%)	0.61

COPD, chronic obstructive pulmonary disease; ICD, implantable cardioverter defibrillator; * according to KDIGO (Kidney Disease—Improving Global Outcomes); ^1^ neurological diseases: apoplex, Restless-Legs-Syndrome; ^2^ hematological diseases: anemia; ^3^ neoplasia: benign prostatic hyperplasia, skin tumor, gingival hyperplasia; ^4^ chronic inflammation: chronic gastritis, reflux esophagitis II, recurrent pancreatitis.

**Table 3:** Flow-cytometric analysis of DC and T_reg_ subsets and MFIs in patients treated with mechanistic target of rapamycin-Inhibitors (mTORI) or calcineurin-inhibitors (CNI).

	mTORI-Group *n* = 19	CNI-Group *n* = 20	*p* Value
DCs/PBMCs [%]	0.68 ± 0.29	0.56 ± 0.28	0.18
BDCA1^+^ mDCs/DCs [%]	26.4 ± 9.5	30.2 ± 9.2	0.21
BDCA2^+^ pDCs/DCs [%]	58.3 ± 11.5	53.3 ± 11.1	0.17
BDCA3^+^ mDCs/DCs [%]	24.2 ± 10.1	27.0 ± 8.4	0.36
BDCA4^+^ pDCs/DCs [%]	79.6 ± 10.6	70.0 ± 27.5	0.16
MFI BDCA1 [U]	16,985 ± 7058	15,550 ± 4204	0.44
MFI BDCA2 [U]	19,205 ± 7504	20,876 ± 7469	0.49
MFI BDCA3 [U]	1803 ± 412	3654 ± 4727	0.10
MFI BDCA4 [U]	2539 ± 1142	2077 ± 612	0.12
CD4^+^ T cells/total T cells [%]	20.7 ± 7.5	22.6 ± 8.4	0.48
T_regs_/total T cells [%]	11.6 ± 3.5	9.0 ± 2.9	0.02
CD120b^+^ T_regs_/total T cells [%]	87.2 ± 8.4	86.7 ± 9.3	0.85
CD147^+^ T_regs_/total T cells [%]	97.7 ± 2.8	97.3 ± 7.7	0.84
CD39^+^ T_regs_/total T cells [%]	35.5 ± 16.3	26.6 ± 10.9	0.05
CD62L^+^ T_regs_/total T cells [%]	86.9 ± 12.5	84.3 ± 9.6	0.48
MFI CD120b [U]	1688 ± 323	1613 ± 214	0.85
MFI CD147 [U]	1769 ± 389	2065 ± 557	0.06
MFI CD39 [U]	783 ± 216	901 ± 307	0.17
MFI CD62L [U]	10,491 ± 2530	9441 ± 2189	0.17

BDCA1/2/3/4, blood dendritic cell antigen 1/2/3/4; CD, cluster of differentiation; DCs, dendritic cells; mDCs, myeloid dendritic cells; MFI, mean fluorescence intensities; pDCs, plasmacytoid dendritic cells; T_regs_, regulatory T cells; U, unit.
